# Progress of Ossification after Mandibular Reconstruction by Free Fibula Flap Depending on Different Timing of Radiotherapy: A Retrospective 3D Analysis by CT Scans

**DOI:** 10.3390/jcm13144104

**Published:** 2024-07-13

**Authors:** Maximilian Gottsauner, Anne Marie Sroka, Jonas Eichberger, Johannes Schuderer, Florian Zeman, Mathias Fiedler, Michael Maurer, Ingo Einspieler, Torsten E. Reichert, Tobias Ettl

**Affiliations:** 1 Department of Oral and Maxillofacial Surgery, University Hospital Regensburg, Franz-Josef-Strauß-Allee 11, 93053 Regensburg, Germany; 2 Department of Prosthetic Dentistry, University Hospital Regensburg, 93053 Regensburg, Germany; 3 Center for Clinical Studies, University Hospital Regensburg, 93053 Regensburg, Germany; 4 Department of Radiology, University Hospital Regensburg, 93053 Regensburg, Germany

**Keywords:** ossification, radiotherapy, microvascular reconstruction, CT, mandible, fibula

## Abstract

**Background:** The aim of this study was to evaluate the difference between pre- and post-operative radiotherapy on the progress of ossification after free fibula flap reconstruction of the mandible using three-dimensional (3D) analysis. **Methods:** A total of 38 free fibula reconstructions of the mandible were evaluated retrospectively for ossification between bone segments by measuring Hounsfield Units (HU) in at least two postoperative computer tomography scans (average of 2.4 scans per patient; around the 5th, 12th, 16th, and 19th month postoperative). Three subgroups were created according to the time of irradiation: preoperative radiotherapy (preORT) (*n* = 11), postoperative radiotherapy (postORT) (*n* = 16), and patients without any radiation therapy (*n* = 11) as the control group (noRT). HU in eight regions of interest (ROI) and overlapping surfaces between segments per contact point, as well as influencing factors, were analyzed. **Results:** The fastest progress in gain of HU ossification with a difference of 0.30 HU/day was observed in noRT compared to preORT (*p* = 0.002). postORT was −0.24 HU/day slower than preORT (*p* = 0.005). Original and grafted bone showed a significantly slower HU uptake than between two graft segments with −84.18 HU/day (*p* < 0.001). Moreover, a larger initial overlapping surface between the segments in cm^2^ resulted in a higher rise of HU/day (*p* < 0.001). **Conclusions:** 3D analysis of post-reconstructive CT scans shows prolonged ossification of mandible reconstructions by free fibula after head and neck radiation. The effect is distinct in cases with post-operative adjuvant radiotherapy. The effects of radiotherapy on ossification may be minimized by a larger initial contact surface and improved operational techniques. Moreover, HU longitudinal measurements and 3D analysis offer new perspectives for clinical evaluation of successful bony healing.

## 1. Introduction

The incidence of oral and oropharyngeal cancer ranks 13th among all cancers for both sexes, according to the IARC Global Cancer Observatory in 2020 [1]. 

Carcinomas invading the mandible normally require jaw resection and postoperative radiotherapy. The indication for adjuvant radiotherapy in minor N0-staged cases is still under investigation [2,3] as is the right timing for mandible reconstruction after resection. Immediate reconstruction is often preferred due to simplified vessel preparation and easier anastomosis [4]. Secondary reconstruction of the mandible enables a reasonable tumor follow up at the expense of post-radiotherapy effects like fibrosis, which can obviate successful microvascular reconstruction [5,6,7]. Definite radiotherapy leading to osteonecrosis of the jaw (ORN), followed by continuity resection and microvascular reconstruction, has similar risk patterns compared to secondary reconstruction after primary surgery and adjuvant radiotherapy [8,9,10].

Other non-cancer-associated severe bony defects of the mandible can emerge from severe maxillofacial traumata, advanced inflammatory osteomyelitis like medication-related osteonecrosis of the jaw (MRONJ), or after resection of benign tumors [11,12].

Microvascular transplants, especially the free fibula flap, have been established as the gold standard for reconstructions of the mandible. Its wide bone of up to 25 cm, the possibility of multi-segmentation and simultaneous harvesting, its reliable and flexible skin paddle, and a long pedicle with a good vessel diameter represent the main advantages of the fibula for mandible reconstruction [13,14,15]. Prior to flap raising, angiography of the lower limb has to be performed to check vascular status [16]. 

**Figure 1:**
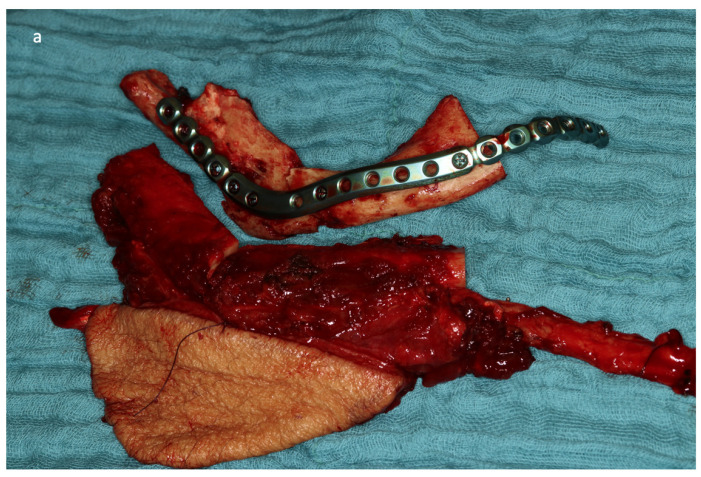

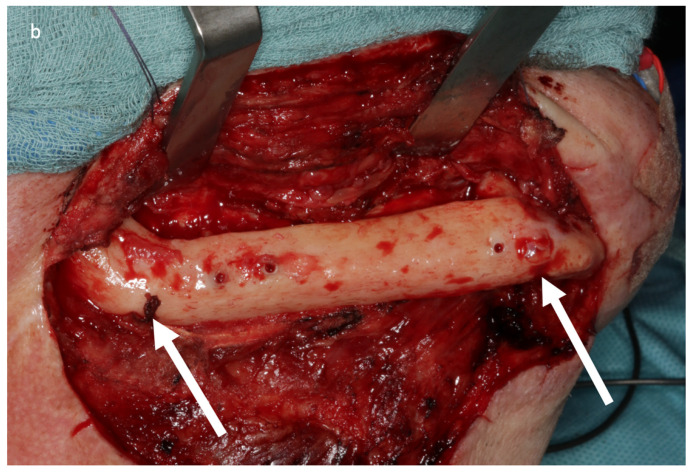
Clinical case of ORN (preORT): reconstruction with two-segmented fibula after hemimandibulectomy: hemimandibulectomy and manually adapted preformed reconstruction plate compared to the harvested two-segmented free fibula with extraoral skin paddle (**a**). After 16 months, there was complete intersegmental ossification in the neo-angle of the mandible and complete ossification between the anterior segment and the original bone in the canine area after the removal of the reconstruction plate (white arrows) (**b**).

Complete and stable ossification between the original bone and transplant as well as between the segments should be achieved for the long-term success of mandibular reconstruction (Figure 1a,b).

This situation is required for complete removal of osteosynthesis hardware and should prevent secondary plate-related complications and simplify dental implantation for full prosthetic rehabilitation [17,18,19]. Another parameter with a possible influence on ossification may be the type of osteosynthesis used for reconstruction of the mandible. A possible benefit of patient-specific implants compared to manually bent plates as well as a comparison between reconstruction plates and mini-plates for effects on bony healing of free fibula flaps are still under discussion [20,21,22]. 

Overall, several studies have shown the feasibility of mandibular reconstruction after radiotherapy with free fibular grafts, although infection rates and wound complications have increased [10,23,24].

The negative effects of radiation on bone healing, cell composition, and bone matrix have already been investigated [25]. Moreover, soft tissue remodeling can lead to severe functional limitations for the patient [26,27].

An analysis of the influence of radiotherapy on the ossification of bony-free flaps has been performed on panoramic X-rays. The results of this two-dimensional (2D) radiographic evaluation showed slower ossification of irradiated free bony grafts compared to the non-irradiated control group without a significant difference between pre- and postoperative radiotherapy. This evaluation of panoramic X-rays provided only subjective results, depending on the investigator’s experience [28]. 

On the other hand, computer tomography (CT) allows an objective measurement of bone density via Hounsfield Units (HU). These correlated histologically with the progress of bone healing [29]. Similar results were reported in another study, where a constant increase in HU during the process of bone healing itself was observed [30].

The aim of the current retrospective study was to objectively re-evaluate the ossification of reconstructed mandibles by free fibula flaps via HU, comparing a non-irradiated control group to pre- and postoperatively irradiated patients using three-dimensional analysis of follow-up computer tomography (CT) scans.

## 2. Materials and Methods

The study design was approved by the ethics committee of the University of Regensburg (ref. 23-3559-104) in accordance with the Helsinki Declaration and its later amendments or comparable ethical standards.

### 2.1. Patients

**Figure A1:**
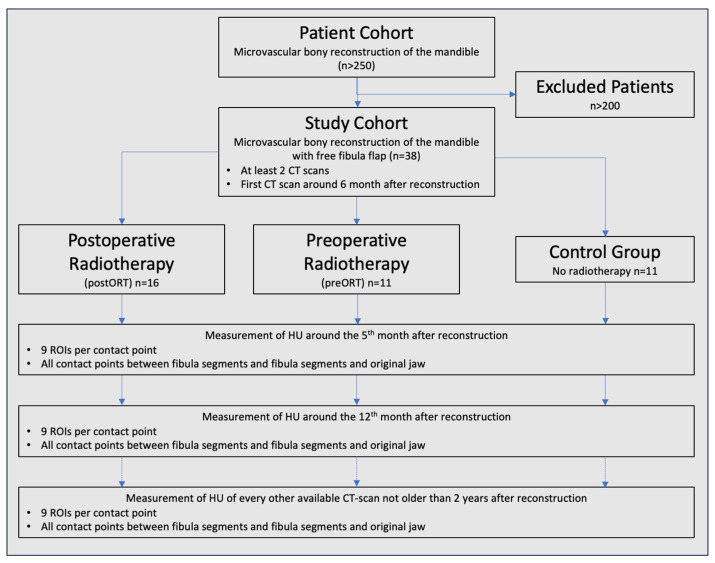
The STROBE Chart shows exclusion, inclusion and analysis of the patient cohort.

Between 2008 and 2022, more than 250 reconstructions of the jaw by free fibula flap were performed in the Department of Oral and Maxillofacial Surgery at the University Hospital in Regensburg. A total of 38 cases fulfilled all of the following criteria: reconstruction of the mandible with a free fibula flap; at least 2 CT scans after reconstruction; and the first CT scan around 6 months after reconstruction. CT scans not older than 2 years after reconstruction were evaluated due to expected changes in the first two years after reconstruction (Appendix A Figure A1). 

The following data were collected: age, gender, diagnosis, radiation history, location of malignancy, preoperative CAD/CAM planning, defect classification after Jewer and Boyd [31], type of bony reconstruction, amount and length of the bone segments, duration on ICU, the duration of hospitalization, additional neck dissection, revisions, smoking, and alcohol abuse. None of the selected patients reportedly received antiresorptive agents.

The cohort was divided into three groups (Table 1): postoperative radiotherapy (postORT) with mandible reconstruction performed before radiotherapy (n = 16); preoperative radiotherapy (preORT) with mandible reconstruction performed after radiotherapy (n = 11); and patients without any radiotherapy (n = 11) as the control group (noRT).

### 2.2. Radiographic Data Management

**Figure 2:**
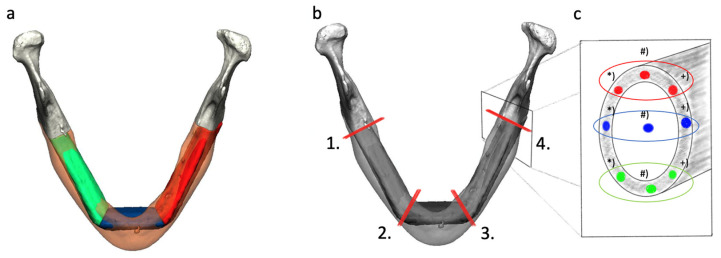
Schematic diagram of Hounsfield Units measurement in the CT scans: Example for CAD/CAM-planned mandible reconstruction with a three-segmented fibula (**a**). Four different contact points (1–4) with a marked plane (red line). There are two contact points between the original bone and the free fibula (one and four) and two contact points between free fibula only (two and three) (**b**). Schematic profile with 9 ROIs: (*) lingual; (#) middle; (+) vestibular and grouped crestal (red), center (blue), and basal (green) (**c**).

**Figure 3:**
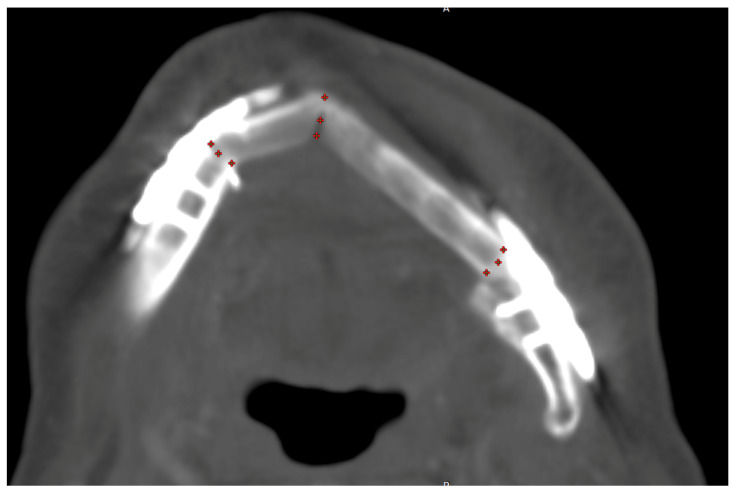
Measurement of Hounsfield Units in the CT scans: measurement of HU in the gap of all contact points with three ROIs each.

All available post-reconstructive CT scans were examined. For staging and follow-up, the radiological department of the university hospital Regensburg used mainly the CT-scanner Somatom Definition Flash (Siemens Healthineers AG, Forchheim, Germany) with standard neck protocol (reconstructed slice thickness 3.00 mm, increment 3.00 mm, kernel Br40) and Accupaque (GE Healthcare GmbH, Düsseldorf, Germany) as weight-adapted contrast media agents (1 mL CM/kg; contrast medium bolus followed by 30 mL NaCl flush; CM delay correlates with CM dose (dose in mL corresponds to delay in sec plus 10 s)). No additional postprocessing techniques for a reduction in beam-hardening artifacts were used. First of all, the CT scan was uploaded to the universal radiological software RadiAnt DICOM Viewer 2023.1 (Medixant, Poznan, Poland). The scan was centered and positioned for measurement in all three layers. In every point of contact, nine regions of interest (ROI of 0.01589 mm^2^) of Hounsfield Units (HU) were quantified in the axial plane (Figure 2), and the mean value of HU was gathered. Three contact points were chosen on each crestal, center, and basal level of the reconstructed mandible (vestibular, middle, and lingual) (Figure 3). After first evaluation, HU values in the middle of the center of the contact point were excluded due to the hollow inside of the fibula bone (Figure 2c; ROI blue #).

Moreover, the overlapping surface between bone segments, the transplanted bone graft, and the original mandible were quantified in square centimeters (cm^2^) by measuring a second ROI in the sagittal contact plane.

**Figure 4:**
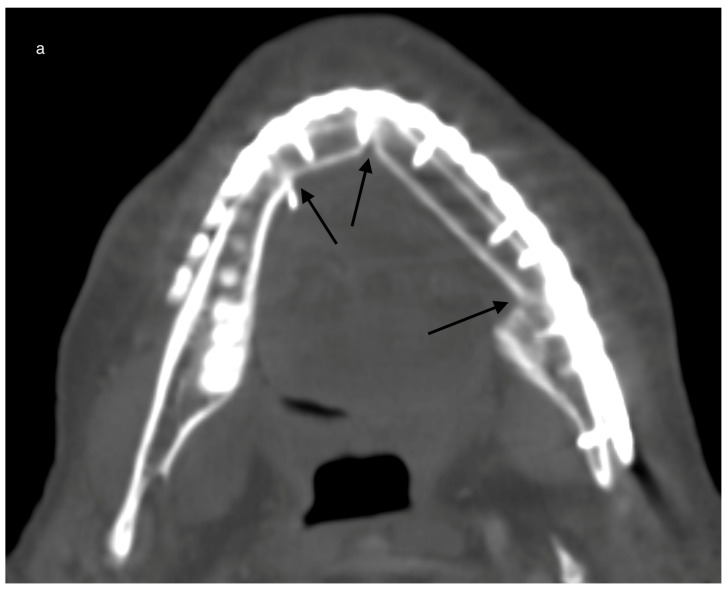

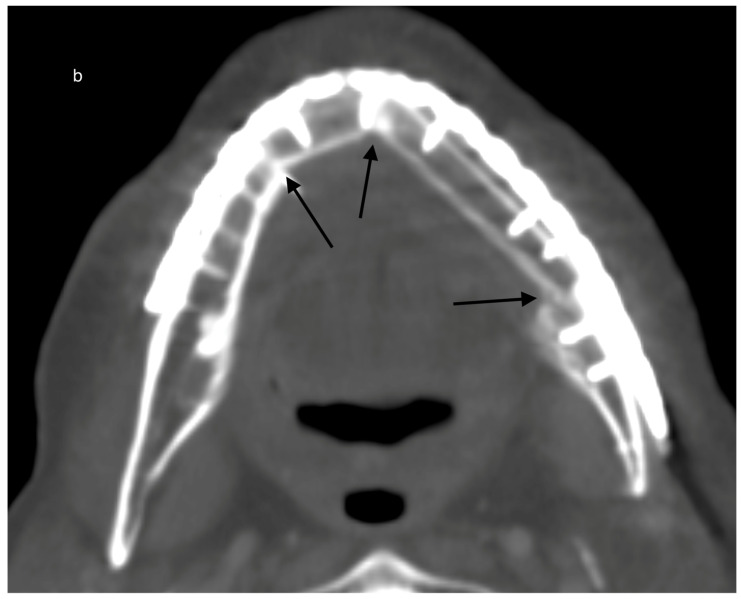
Example for ossification in CT scans over time in a case with a primary reconstructed mandible after malignant tumor resection and post-reconstructive adjuvant radiotherapy (postORT): Example for a reconstructed mandible with a 2-segmented fibula 265 days after operation. All three contact points are marked with black arrows (**a**). Slow progression of ossification (black arrows) after 494 days (**b**).

All HU measurements were repeated for every follow-up CT scan (Figure 4).

### 2.3. Data Analysis

Continuous data are presented as the mean ± SD. A linear mixed model was used to analyze whether the increase in HU differed over time between the three groups. Therefore, HU was included as the dependent variable, while time in days between the follow-up measurements, group (preOR, postOR, and the control), and the interaction term of time in days and group were added as independent variables. The model was further adjusted for location in XXX direction (basal, cranial, middle), location in YYY direction (vestibular, lingual, middle), contact bone (between segments; contact to original bone), and diameter of CP gap. The structure of the covariance matrix of the repeated measurements over time was set to “unstructured”. A random intercept was defined for the patient, for the location of measurement nested within the patient, and for the location of the contact point nested within the location of measurement. Estimated marginal means with corresponding 95% confidence intervals are presented as effect estimates. All analyses were performed using SAS 9.4, and the procedure was mixed. A *p*-value < 0.05 was considered statistically significant.

## 3. Results

### 3.1. Cohort Characteristics

Overall, 93 CT scans were analyzed. The first CT scans (n = 38) were performed on an average of 139 ± 88 days (approx. 5 months) after reconstruction of the mandible. The second scan (n = 38) took place around the 12th month (mean: 365 ± 173 days) and the third scan (n = 13) around the 16th month (mean: 467 ± 156 days). The last CT scans (n = 4) were carried out around the 19th month (mean: 576 ± 101 days). A total of 1899 ROIs for HU measurements were taken into account. 

In 100% (n = 16) of cases in the postORT subgroup, reconstruction was combined with neck dissection, compared to 55% (n = 6) in the preORT subgroup and 82% (n = 9) in the control group. 

Operation time was an average of 563 ± 205 min longer in the postORT subgroup than in the preORT subgroup (mean: 477 ± 124 min) and the control group (mean: 514 ± 121 min), showing no significant difference between the groups (*p* = 0.404). 

The postoperative stay on the ICU after reconstruction also showed no significant difference (*p* = 0.988): postORT subgroup 4.3 ± 2.7 days, preORT subgroup 4.2 ± 2.6 days, and noRT group 4.1 ± 2.8 days. Concerning overall hospitalization, the postORT subgroup (mean: 16.7 ± 9.4 days), the preORT subgroup (mean: 15.8 ± 7.7 days), and the control group (mean: 12.9 ± 6.3 days) revealed no significant difference (*p* = 0.490). 

The parameter for defect size in the postORT subgroup had the largest average defect with 99.2 ± 29.1 mm, followed by the preORT subgroup with 97.0 ± 29.4 mm and the control group with 91.8 ± 23.7 mm.

### 3.2. HU Measurement

Overall, HU at the contact points increased highly significantly (*p* < 0.001) over time after reconstruction of the mandible. The average gain in HU was 0.34 [0.21, 0.47] per day.

The starting point concerning HU showed no significant difference (*p* = 0.804) between the three groups. 

The increase in HU over time within the groups differed significantly (*p* < 0.001). The average increase in HU per day of the control group (noRT) was 0.30 [0.11, 0.48] HU higher compared to the preORT group (*p* = 0.002), while the preORT group showed a higher increase in 0.24 [0.08, 0.41] HU per day compared to the postORT group (*p* = 0.005). 

Moreover, contact points between fibula segments and the original bone of the mandible had a smaller increase of −84.18 [−126.13, −42.22] HU per day compared to contact points between fibula segments only (*p* < 0.001).

Analyzing the overlapping surface in the contact zone, an enhancement of 1cm^2^ would lead to a boost of 107.86 [53.08, 162.64] HU per day (*p* < 0.001).

Grouping the locations of ROIs and their measurements leads to different significant results determined by the orientation of the grouping (Table 2). The subdivisions of the ROIs for measurement at the contact point horizontally and vertically are explained in Figure 2c.

## 4. Discussion

The timing for bony reconstruction of the mandible under the influence of radiotherapy has been controversially discussed. Resection of advanced malignancies and immediate microvascular reconstruction of the mandible by free fibula flaps are established procedures in various treatment centers [32]. The alternative treatment after mandibulectomy is a primary alloplastic reconstruction with stable osteosynthesis. Additional soft tissue transfers, such as anterolateral thigh flaps, may be necessary to minimize the risk of plate exposure and wound dehiscence, especially in combination with radiotherapy [33,34]. Radiotherapy also has a major impact on the donor vessels in the head and neck region, often leading to fibrosis. These fibrotic vessels with a breakdown of the intima lining lead to challenging situations for delayed secondary microsurgery [4,5]. With regard to the drawbacks of primary alloplastic reconstruction—and also from our own experiences—primary microvascular bony reconstruction seems to be the treatment of choice and outweighs the impaired bony healing due to adjuvant radiotherapy [35]. 

The effects of radiotherapy on the ossification of bony microvascular reconstructions of the jaw have already been under investigation [28]. The previous study was based on panoramic radiographs and evaluated ossification by using a subjective score (no ossification, partial ossification, and complete ossification). In contrast, the current study objectively analyzes bony ossification of FFFs for mandibular reconstruction by measuring Hounsfield-Units in ROIs of postoperatively performed CT scans, which is much more exact. Moreover, measuring diameter at the contact point as well as an analysis of the longitudinal gain of HU may lead to a better prediction of the process of ossification of microvascular reconstruction in the future.

Hounsfield units in different anatomical regions are already used for detecting bone density without any further calibration [36,37]. Moreover, HU and bone density correlate with age and sex [38]. Preclinically, in the longitudinal follow-up of bone healing, measuring HU in a circumscribed ROI has shown a constant increase in HU, especially for the denser cortical bone [39].

Comparing the current results with the results of the study on panoramic radiographs, a significant difference in the longitudinal gain of HU could be seen between all subgroups, especially between preORT and postORT. In panoramic radiographs, the difference between preORT and postORT has shown only a tendency without significance (*p* = 0.087) [28]. Analysis of HU confirmed the faster ossification between fibula segments in contrast to the bone healing between the bones of the mandible and the free flap. The disparity between preORT and postORT could be derived from faster ossification between the non-irradiated fibula segments in the preORT subgroup compared to the ossification of the irradiated fibula in the postORT group. The quality of initial bone contact seems to be another key factor in accelerating bone healing. The idea of enlarging the contact surface between bone segments in order to support ossification is confirmed in this study by the 3D measurement of the CT scan and the progression of HU [28].

Akashi et al. evaluated the bone union of reconstructed fibula flaps with panoramic x-rays as well as with CT scans. An influence of radiotherapy on the bone union could not be proven in the small cohort of 20 patients, 2 with preoperative and 5 with postoperative radiotherapy. Non-union was only found in the angle of the mandible. CT scans were only evaluated with a subjective score. After a 2-year follow-up, they reported an overall of 9% radiographic non-unions in the reconstructed mandibles. The angle may be more problematic due to a smaller bone contact surface between the thin original mandibular bone in this area and the wider round profile of the transplanted fibula.

The predictive value of initial bone contact was investigated by Swendseid et al. A larger gap of 1 mm in CT scans was combined with a higher risk of non-union. Ossification was evaluated subjectively by raters in this study. Cancer diagnosis, in contrast to pre- or postoperative radiation, showed a significant risk for non-union of microvascular reconstructions of the jaw [40].

A volumetric analysis of gaps between segments by cone-beam computer tomography (CBCT) could not show a significant difference between the different bony contact points of a free fibula flap reconstruction. There were no differences between fibula-fibula contact and mandible-fibula contact. Beside wound healing disorders, adjuvant radiotherapy was a significant negative predictive parameter for the bony union of the reconstruction and confirmed the unfavorable impact of radiation on bone healing [41]. Because CBCT is only capable of measuring gray values instead of HU, an analogous measurement could not be performed.

The influence of mini-plates versus reconstruction plates on ossification of free fibula flaps cannot be judged due to missing mini-plate cases in the current study [21].

As a limitation of this study, no distinct HU value could be provided for specific time points due to the irregular timing of CT scans. Moreover, no target value or specific threshold for successful ossification could be defined, given the intra- and interindividual differences in bone density. Of note, beam-hardening artifacts from the reconstruction plate can severely affect HU measurements [42]. However, the longitudinal analysis of the quantification of 9 ROIs at each contact point should have a minimizing effect on the statistical results.

The protocol used in the study is based on a soft reconstruction kernel. Reconstruction of the images with a hard kernel was not possible due to the lack of raw data.

The statistical model scales up the changes in HU of the contact point on a daily basis because the provided data (period between bony reconstruction and every single CT scan) is based on days-between-events. The intention of this study is more likely to evaluate the progress of ossification in a long-term context. In the future, the information provided by two CT scans in a 3- to 6-month interval may help to forecast the further progress of ossification. If necessary, monthly changes in HU can be evaluated by multiplying the results, for example, by 30.5 without statistical changes.

The results of the current study may conclude that creating better contacts between segments leads to faster ossification. In this context, a systematic review with meta-analysis recently compared computer-aided design and manufacturing with conventional surgical planning for head and neck reconstruction [43]. Although not evaluating ossification in detail—as performed in this study—the results interestingly did not reveal a significantly reduced rate of non-unions by using CADCAM surgery, as one might assume. Nevertheless, the non-union rate in the CADCAM group was lower (22.9%) compared to the conventional group (32.2%). Beside individual CAD/CAM reconstruction planning [44], partly-adjustable resection and cutting guides [45] or geometric cuts by robotic laser osteotomy [46] may improve the accuracy between bone segments.

## 5. Conclusions

The current study demonstrates the unfavorable effects of radiotherapy on the ossification of free fibula flaps. Post-reconstructive adjuvant radiotherapy decelerates ossification significantly, even more than pre-reconstructive radiotherapy. Non-irradiated free fibula flaps showed the fastest ossification. Post-surgical CT scans can objectively demonstrate the longitudinal course of ossification with the use of Hounsfield Units. Technical considerations aiming for a larger overlapping contact surface between the bone segments may improve ossification and balance the negative impact of radiation on bone healing. The longitudinal measurement of HU at the contact point for predicting the progress of ossification may be an additional tool for clinical evaluation of microvascular bony reconstruction in the future.

## Figures and Tables

**Table 1:** Cohort characteristics for all three subgroups.

	Postoperative Radiotherapy (n = 16)	Preoperative Radiotherapy (n = 11)	Non-Irradiated (Control Group) (n = 11)
Male/female, n (%)	12 (75%)/4 (25%)	7 (64%)/4 (36%)	6 (55%)/5 (45%)
Age in years, median (IQR)	61 (56, 69)	60 (50, 67)	61 (57, 65)
Radiation dose in Gy, median (IQR)	64.4 (60.0, 66.6)	66.0 (60.0, 72.0)	none
Tobacco, n (%)	11 (69%)	5 (46%)	7 (64%)
Alcohol, n (%)	9 (56%)	3 (27%)	4 (36%)
Revisions, n (%)	9 (56%)	2 (18%)	5 (46%)
CAD/CAM planning, n (%)	none	4 (36%)	1 (9%)
Neck dissection, n (%)	16 (100%)	6 (55%)	9 (82%)
Primary Diagnosis			
Malignant tumor	16	8	9
Osteoradionecrosis	0	3	0
Unspecific osteomyelitis	0	0	1
Gun shot wounds	0	0	1
Side of malignancy (n = 33) n = 30 cases of oral squamous cell carcinoma or its metastases with (suspected) infiltration of the mandible/ n = 1 case of high-grade soft tissue sarcoma/ n = 1 case of ameloblastic carcinoma of the mandible/ n = 1 case of oral malignant melanoma			
Lateral floor of mouth/mandible	14	3	8
Multiple locations	2	5	1
Side of primary malignancy in ORN (n = 3)			
Tongue		1	
Pharynx/tonsil/root of tongue		1	
Larynx		1	
Defect Classification of Boyd and Jewer			
C	0	0	1
H	0	3	0
HCL	0	1	0
L	8	4	2
LC	2	2	5
LCL	6	1	3

Revisions were smaller postoperative procedures like revision of microvascular anastomosis, excision of fistulas, or closure of dehiscence. IQR, interquartile range; CAD/CAM, computer-aided design/computer-aided manufacturing; ORN, osteoradionecrosis.

**Table 2:** Linear mixed model estimating the average HU baseline, group, and location. The gain of HU for comparisons is calculated only in relation to the reference without absolute values.

	Estimate	95%-CI		*p*-Value
HU per Group at baseline (*p* = 0.804)				
preORT	reference			
noRT (control group)	−52.90	−210.17	104.38	0.510
postORT	−28.73	−173.38	115.92	0.697
HU gain overall for grouping locations horizontally #1 per day (*p* = 0.003)				
basal	reference			
cranial	5.26	−53.34	63.86	0.860
middle	114.06	45.35	182.76	0.001
HU gain overall for grouping locations vertically #2 per day (*p* = 0.005)				
Vestibular (+)	reference			
Lingual (*)	−98.72	−157.31	−40.13	0.001
Middle (#)	−86.70	−155.41	−17.99	0.013

Colors in HU gain for grouping locations horizontally refer to Figure 2c. Letters in HU gain for grouping locations vertically refer to Figure 2c.

## Data Availability

Data can be obtained by scientists that conducted the work independently from the industry, on request to the corresponding author. Data are not stored on publicly available servers and contain patient ID.
